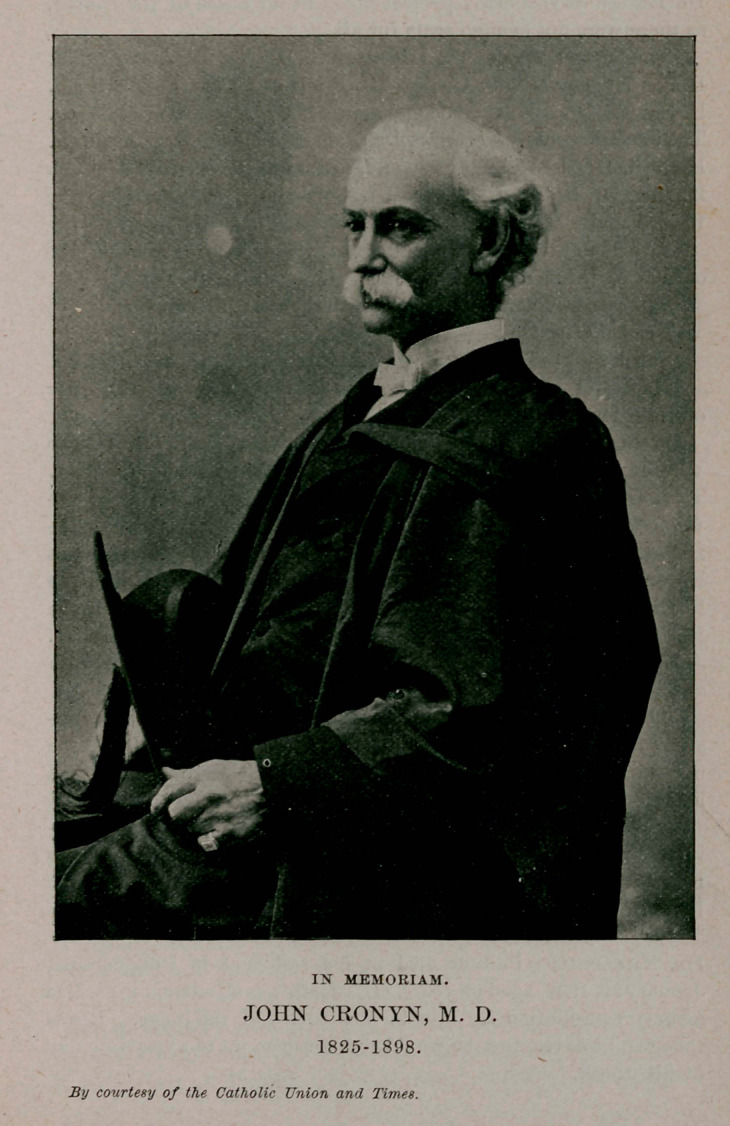# Dr. John Cronyn

**Published:** 1898-03

**Authors:** 


### Obituary.

DR. JOHN CRONYN, of Buffalo, died at his home in this city, No. 55 West Swan street, at 6 o'clock Friday evening, February 11, 1898, aged 72 years. He was stricken with paralysis on Monday night previous to his death, and though he rallied once or twice during the week, fatal symptoms supervened two days before his death and he gradually sank, becoming unconscious a few hours before his death. He died surrounded by his family and medical attendants.

Dr. Cronyn was born December 15, 1825, at Black Rock, a suburb of Cork, Ireland. He received his preliminary education in a school at Cork, under the supervision of his father and after he came to America was placed in charge of private tutors in Knox's college, Toronto. He came to this country in 1837 in company with his widowed mother, five brothers and one sister, and located at Toronto. In a few years he began the study of medicine, which he pursued in the University of Toronto, and passed his examinations for the degree of M. D. in 1850. He did not receive his diploma, however, until a few years later, when the Canadian government removed all sectarian restrictions. He then applied for and obtained his medical degree, and for his thesis was awarded the chancellor's prize. He first located for practice at Fort Erie, where for several years he was local superintendent of schools and reeve of that place. In 1859, Dr. Cronyn came to Buffalo and established himself at the corner of Church and Pearl streets. He rapidly gained an active professional practice and soon was appointed first as surgeon and next as physician-in-chief of the medical staff of the Buffalo Hospital Sisters of Charity, which latter office he held until his death. The medical department of Niagara University was established in 1883 largely if not principally through his instrumentality, and in that college he held the chair of principles and practice of medicine, and was president of the medical faculty from the foundation of the school until his end. In 1888, Niagara University conferred upon him the degree of Ph. D., and in 1893 that of LL. D.

Dr. Cronyn was president of the New York state medical association (1888), twice president of the medical society of the county of Erie (1875–1876), twice president of the Buffalo medical and surgical association (1876–1883) and an honorary member of the Ontario medical association. For several years he was a member of the board of managers of the Buffalo state hospital and a part of the time served as president of the board.

Dr. Cronyn was a literal student of medicine, well versed in professional lore, easy and ready in debate, an excellent teacher and a courteous gentleman. He acquired a very large practice, was often called long distances as a consultant and justly achieved a wide fame as a family physician. He had a love for music, art and religion, and died a consistent believer in the Roman catholic faith. His funeral services were held in St. Joseph's cathedral, Monday, February 14, 1898. The remains was escorted from the Cronyn home to the cathedral by members of the faculty of Niagara University and the medical society of the county of Erie. The students of Niagara University stood in a double line at the

IN MEMORIAM.

JOHN CRONYN, M. D.

1825-1898.

*By courtesy of the Catholic Union and Times.*

portico of the cathedral and the casket was borne between them. During the service many persons stood in the aisles of the church, as there were not enough seats for all.

The bearers were : Dr. Lawrence G. Hanley, Dr. Joseph P. F. Burke, Dr. Edward M. Dooley, Dr. Francis J. Carr, Dr. David L. Redmond, Dr. Sidney A. Dunham and Dr. Frank W. Maloney. The honorary bearers were : Dr. Thomas Lothrop, Dr. Alvin A. Hubbell, Dr. Henry D. Ingraham, Dr. Herman Mynter, Dr. William H. Pitt, Dr. Rollin L. Banta, Dr. Cornelius C. Wyckoff and the Hon. Thomas V. Welch, of Niagara Falls.

A solemn requiem was celebrated, the following being the officers of the mass :

Celebrant—The Rev. John D. Biden, rector of the cathedral.

Deacon of the mass—The Rev. T. P. Lynch.

Sub-deacon—The Rev. P. J. Enright.

Master of ceremonies—The Rev. John J. Sheehan, chancellor of the diocese.

Within the sanctuary were the following clergymen : The Very Rev. M. P. Connery, vicar-general of the diocese ; the Rev. Thomas Donoghue, D. D. ; the Rev. Patrick Cronin, editor of the *Catholic Union and Times* ; the Rev. P. Hoelscher, D. D.; the Rev. Luke A. Grace, D.D., of Niagara University ; the Rev. James F. Mooney ; the Rev. L. Bonvin, S. J., of Canisius college ; the Rev. Father Hickey, C. M.; the Rev. J. O. M. Hayden, C. M.; the Rev. Daniel L. Walsh of the Working Boys' Home ; the Rev. M. J. Keane, the Rev. John J. Sheehy, the Rev. Jeremiah McGrath and the Rev. L. Martinelli.

The Very Rev. P. McHale, C. M., president of Niagara University, preached the funeral sermon. He spoke eloquently of the many virtues of the deceased physician and impressed on his hearers the deep loss the community has sustained.

The burial was at Holy Cross Cemetery. Dr. Cronyn is survived by a widow and six children. The children are the Misses Elizabeth, Jennie, Juliana and May, and Thomas and Dr. John L. C. Cronyn.

## Figures and Tables

**Figure f1:**